# Implant Stability in Narrow Posterior Alveolar Ridges: A Randomized Controlled Trial Comparing Conventional Ridge Split and Osseodensification

**DOI:** 10.7759/cureus.86723

**Published:** 2025-06-25

**Authors:** Meghna D Agarwal, Girija Dodamani, Arun Dodamani, Suresh Nagaral, Sunil Ronad, Ashwini Pungle, Seema Gupta

**Affiliations:** 1 Department of Prosthodontics, Jawahar Medical Foundation's Annasaheb Chudaman Patil Memorial Dental College, Dhule, IND; 2 Department of Public Health Dentistry, Jawahar Medical Foundation's Annasaheb Chudaman Patil Memorial Dental College, Dhule, IND; 3 Department of Prosthodontics, Dayananda Sagar College of Dental Sciences, Bengaluru, IND; 4 Department of Orthodontics, Kothiwal Dental College and Research Centre, Moradabad, IND

**Keywords:** conventional, dental implants, mandibular, narrow, ridge, stability

## Abstract

Introduction: Successful implant placement in the posterior mandible often requires augmentation of narrow ridges. This study aimed to compare the primary stability of dental implants placed using the conventional ridge split technique and the osseodensification method in narrow posterior mandibular ridges.

Methods: A randomized controlled trial was conducted involving 14 patients, with a total of 28 implants divided equally between two groups. Group I underwent conventional ridge splitting, and Group II received implant site preparation via osseodensification. Primary stability was evaluated immediately after implant placement using the Osstell Mentor device (Osstell AB, Gothenburg, Sweden) to obtain implant stability quotient (ISQ) values. Data was analyzed, and mean ISQs were compared using independent t-tests.

Results: Implants placed using the osseodensification technique exhibited significantly higher ISQ values compared to those placed using the conventional ridge split technique (p < 0.001). No significant difference in stability was observed between the lower first and second molar sites bilaterally within each group. The results indicated that osseodensification offered superior primary stability.

Conclusion: Both the conventional ridge split and osseodensification techniques effectively enabled implant placement in narrow posterior mandibular ridges. However, the osseodensification technique demonstrated significantly greater primary stability, suggesting its potential advantage in clinical practice for achieving predictable outcomes in compromised ridge scenarios.

## Introduction

Implant dentistry has evolved significantly, with modern restorative materials and surgical techniques providing predictable solutions for tooth replacement [1]. Implant-supported crowns represent a well-established treatment option for partially edentulous patients, offering both functional rehabilitation and aesthetic restoration. A critical challenge in posterior mandibular implant placement involves managing the alveolar ridge atrophy that commonly follows tooth extraction, often resulting in insufficient bone width for optimal implant positioning [2].

Clinical evidence suggests that a minimum ridge width of 8 mm is required for standard-diameter implant placement without compromising buccal plate integrity. Post-extraction resorption frequently reduces ridge dimensions to 4 mm or less, creating anatomical limitations that may lead to implant dehiscence or perforation if not properly addressed [3]. This anatomical constraint necessitates ridge augmentation procedures to achieve prosthetically driven implant placement. Current treatment modalities for horizontal ridge deficiency include guided bone regeneration, autogenous block grafting, alveolar distraction osteogenesis, and ridge expansion techniques [4].

The ridge expansion technique enables lateral alveolar ridge augmentation with simultaneous implant placement. While clinically effective, this approach presents potential complications including cortical plate fracture and compromised healing, particularly in cases with poor bone quality [5,6]. In contrast, osseodensification represents a contemporary osteotomy preparation method that differs fundamentally from conventional subtractive drilling [7]. This technique employs specialized burs operating in a non-cutting, counterclockwise rotational mode to preserve and condense bone along the osteotomy walls. The resulting increase in bone density enhances primary implant stability while maintaining native bone volume, demonstrating particular efficacy in low-density bone sites [8,9].

Primary implant stability remains a fundamental prerequisite for successful osseointegration and long-term implant survival, influenced by multiple variables including bone quality, surgical protocol, and implant design characteristics [10]. Clinical assessment of primary stability typically involves insertion torque evaluation or resonance frequency analysis using devices such as the Osstell system, which quantifies stability through implant stability quotient (ISQ) measurements [11,12]. Recognizing the clinical significance of achieving optimal primary stability in narrow ridge situations, this randomized controlled trial was designed to compare primary stability outcomes between conventional ridge split and osseodensification techniques in the atrophic posterior mandible.

## Materials and methods

Study design and setting

This randomized controlled clinical trial was conducted in the Department of Prosthodontics, Jawahar Medical Foundation's Annasaheb Chudaman Patil Memorial Dental College, Dhule, India, in accordance with the Consolidated Standards of Reporting Trials (CONSORT) guidelines from November 2024 to March 2025. Ethical clearance was obtained from the Institutional Ethics Committee (IEC) of Jawahar Medical Foundation's Annasaheb Chudaman Patil Memorial Dental College, Dhule, Maharashtra, India, under approval number EC/NEW/INST/2022/2959/2022/060. This study was registered with the Clinical Trial Registry of India (CTRI/2024/11/076283). All participants were thoroughly informed about the nature of the study, and written informed consent was obtained prior to enrolment. This study was conducted in accordance with the principles of the Declaration of Helsinki.

Sample size calculation

The sample size was calculated using G Power software version 3.1.6 (Heinrich-Heine-Universität Düsseldorf, Düsseldorf, Germany), determining a minimum of 14 participants (seven per group) based on an a priori t-test for differences between two independent groups. The calculation assumed a study power of 80%, an α error of 0.05, and an effect size of 1.4, derived from a study by Elgrany et al. [3], which evaluated ISQ measures.

Patients' selection

The study included 14 patients with edentulous posterior mandibular segments requiring implant placement. The inclusion criteria were good oral hygiene, presence of at least two missing teeth in the posterior mandible (first or second molars on either side), minimum crestal ridge width of 3 mm, and minimum bone height of 12 mm from the ridge crest to the superior border of the mandibular canal. Only sites with D1 or D2 bone quality were included in the study. The exclusion criteria included systemic conditions affecting healing, corticosteroid use, smoking, and parafunctional habits such as bruxism.

Randomization

Participants were randomly allocated to two groups (Group I: conventional ridge split technique; Group II: osseodensification) using a computer-generated randomization list created via randomizer.org. The randomization process employed a simple randomization method with a 1:1 allocation ratio to ensure an equal number of implant sites (seven per group) in each arm. The randomization sequence was generated using a coin-flipping simulation on randomizer.org, where each "flip" determined assignment to either Group I or Group II, ensuring an unbiased and unpredictable allocation. The randomization was stratified by the anatomical region (mandibular first molars (36/46) or second molars (37/47)) to balance the distribution of implant sites across these regions between groups. This stratification minimized potential confounding effects related to anatomical differences.

Allocation concealment

To prevent selection bias, allocation concealment was implemented using sequentially numbered, opaque, sealed envelopes. After generating the randomization sequence, an independent researcher, not involved in participant recruitment or treatment, prepared the envelopes. Each envelope contained the group assignment (Group I or Group II) for a specific participant and was labelled with a unique participant identification number. These envelopes were stored securely and opened only at the time of implant site allocation, immediately before the procedure, by a designated study coordinator. This process ensured that neither the clinicians performing the procedures nor the participants were aware of the group assignment until the moment of intervention, maintaining the integrity of the randomization process.

Blinding

Given the nature of the surgical interventions (conventional ridge split technique versus osseodensification), blinding of the operating clinicians was not feasible, as the techniques required distinct procedural steps and instrumentation. However, patient blinding was implemented to minimize performance bias. Patients were not informed of their group assignment, and both procedures were performed under similar clinical settings (e.g., same operatory, anesthesia protocols, and post-operative care) to maintain consistency. Outcome assessors, including those evaluating outcomes, were blinded to the group assignments. Data collectors and statisticians analyzing the results were also blinded, with group assignments coded as “A” or “B” in the dataset to prevent bias during analysis. Blinding was maintained throughout the study until the final data analysis was completed. It was a single blind study.

Pre-operative assessment and medication protocol

Pre-operative evaluation consisted of clinical and radiographic assessment, including intraoral periapical radiographs and cone-beam computed tomography (CBCT), to measure bone dimensions and assess anatomical structures. Prophylactic antibiotics were initiated 24 hours prior to surgery and continued for five days postoperatively. Amoxicillin 2 g orally, one hour prior to surgery, as a single preoperative dose, was administered for all patients. The regimen with amoxicillin-clavulanate 625 mg twice daily and metronidazole 400 mg three times daily was extended for five days postoperatively. For penicillin-allergic patients, azithromycin 500 mg orally one hour before surgery was prescribed. The strict oral hygiene with 0.12% chlorhexidine mouthwash pre- and postoperatively was maintained.

Surgical protocol

**Figure 1:**
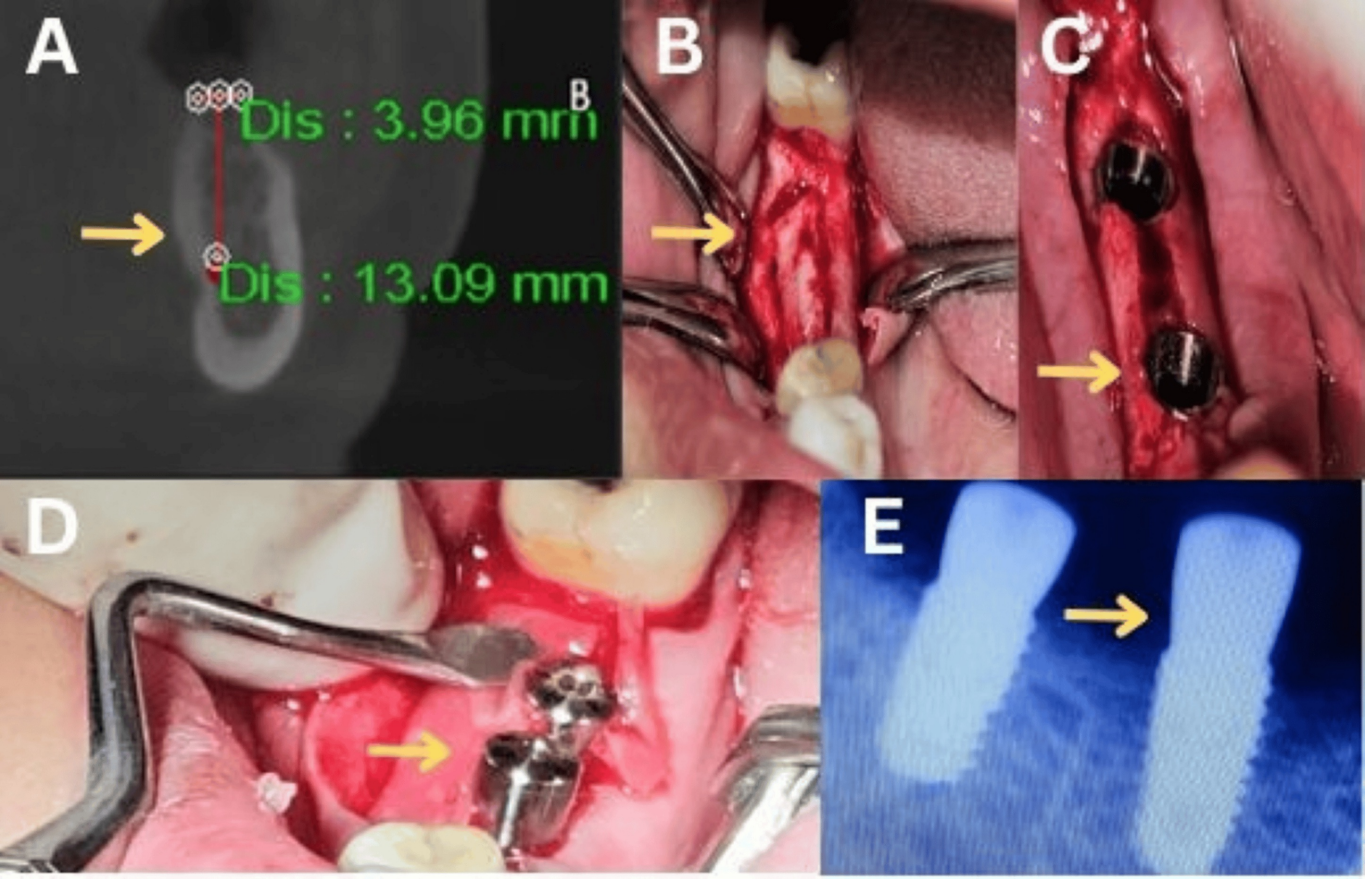
Conventional ridge split technique. (A) Pre-operative cone-beam computed tomographic measurements of the mandible at the lower right first molar region (yellow arrow). (B) Horizontal and vertical incisions in the alveolar crest (yellow arrow). (C) Implant placement (yellow arrow). (D) Healing abutment placement (yellow arrow). (E) Post-implant intraoral periapical radiograph of the lower right first and second molars (yellow arrow). This figure represents intraoral pictures of a patient from the study, and was published with the patient's consent.

In Group I (conventional ridge split technique), a piezoelectric unit (Piezotome Solo, Acteon Group, Mérignac, France) was used to perform a horizontal crestal cut along with two vertical cuts. A full-thickness mucoperiosteal flap was elevated, and the ridge was gradually expanded before implant placement (Figure 1).

**Figure 2:**
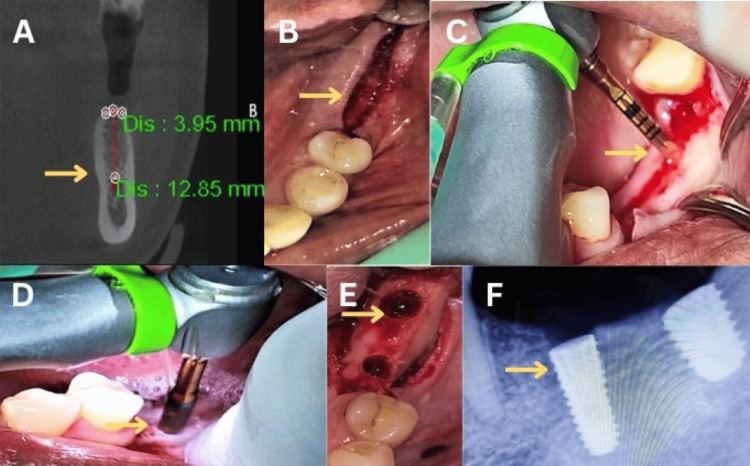
Osseodensification technique (A) Pre-operative cone-beam computed tomographic measurements of the mandible at the lower left first molar region (yellow arrow). (B) Mucoperiosteal incision in the alveolar crest (yellow arrow). (C) Pilot drilling (yellow arrow). (D) Densah bur drilling (yellow arrow). (E) Bur hole for implant placement (yellow arrow). (F) Post-implant intraoral periapical radiograph of lower left first and second molars (yellow arrow). This figure represents intraoral pictures of a patient from the study, and was published with the patient's consent.

In Group II (osseodensification technique), initial osteotomy was performed using a pilot drill at 1200 rpm in a clockwise direction. Site preparation was completed using Densah burs (Versah LLC, Jackson, MI, USA) in a counterclockwise, non-cutting direction (densifying mode), and the osteotomy was incrementally enlarged to the required diameter (Figure 2).

Implant placement and primary stability assessment

**Figure 3:**
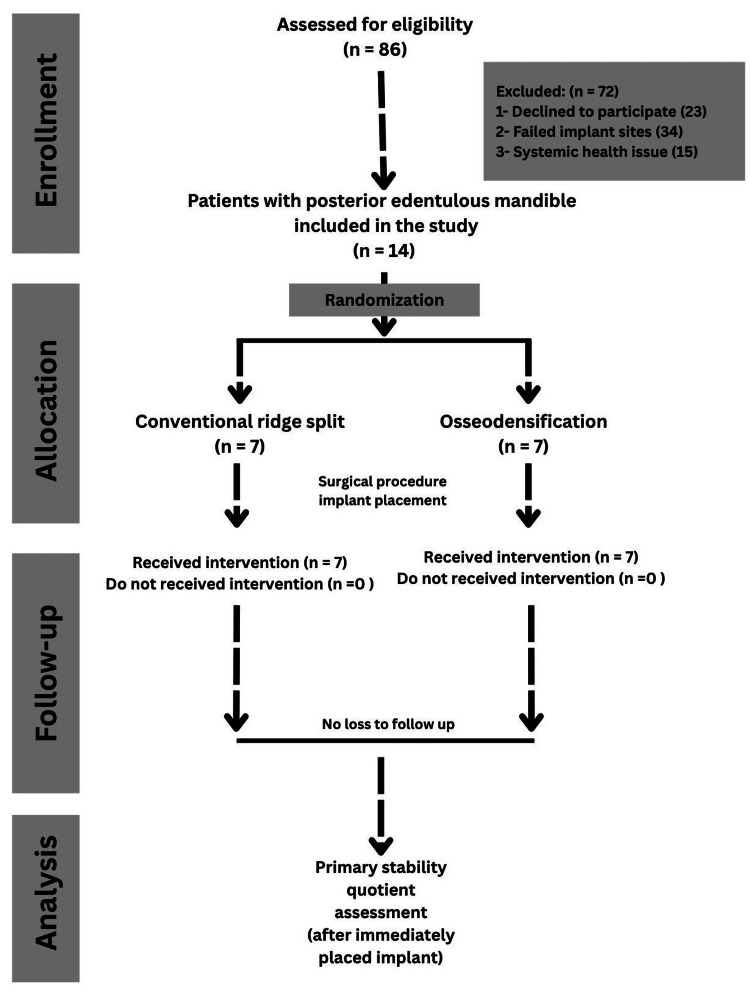
CONSORT flowchart of the study. CONSORT: Consolidated Standards of Reporting Trials

Osstem implants (Osstem Implant Co., Ltd., Seoul, Korea) were used in both groups. All implants were placed in an appropriate alignment. Primary stability was assessed immediately after placement using an Osstell Mentor device (Osstell AB, Gothenburg, Sweden), which measured the resonance frequency and calculated the ISQ. The measurements were recorded in the buccolingual direction. The study protocol followed the CONSORT guideline as depicted in Figure 3.

Postoperative care and prosthetic loading

Postoperative care included the application of cold compresses, the use of 0.12% chlorhexidine mouthwash, and strict oral hygiene. The patient received amoxicillin-clavulanate (625 mg twice daily) and metronidazole (400 mg three times daily) for five days. Pain was managed with diclofenac potassium (50 mg three times daily). The implants were loaded after a three-month healing period, following radiographic confirmation of osseointegration.

Statistical analysis

Data were analyzed for normal distribution using the Shapiro-Wilk test and were found to be normally distributed. An independent t-test was performed to evaluate differences in ISQ values between the two groups and between the 36/46 and 37/47 regions. Statistical significance was set at p < 0.05. All tests were performed using the IBM SPSS Statistics for Windows, Version 26 (Released 2020; IBM Corp., Armonk, New York, United States).

## Results

Table 1 compares primary ISQ between osseodensification and conventional ridge split techniques for teeth 36/46 and 37/47. For teeth 36/46, the osseodensification group exhibited a mean ISQ of 70.57 ± 6.40, which was significantly higher than the ridge split group’s mean of 46.14 ± 2.79, with a mean difference of 24.43 (p = 0.001). Similarly, for teeth 37/47, osseodensification yielded a mean ISQ of 69.43 ± 6.37, compared to 46.14 ± 2.97 for ridge split, with a mean difference of 23.29 (p = 0.001). These findings indicated that osseodensification consistently achieved higher primary implant stability than the conventional ridge split technique, with statistically significant differences (p < 0.05) in both tooth groups. Osseodensification may offer a more reliable method for enhancing implant stability, potentially improving long-term implant success rates in the mandibular posterior region.

**Table 1:** Comparison of primary implant stability (implant stability quotient as ISQ) between osseodensification and conventional ridge split techniques using independent t-test. * p < 0.05: significant, 36/46: mandibular right and left first molar teeth, 37/47: mandibular right and left second molar teeth. Data is presented in the form of mean ± standard deviation (SD).

Tooth region	Techniques	Mean ± SD	Range	Mean difference	t value	p-value
36/46	Osseodensification	70.57 ± 6.40	61-82	24.43	9.256	0.001*
	Conventional ridge split	46.14 ± 2.79	41-50			
37/47	Osseodensification	69.43 ± 6.37	61-80	23.29	8.763	0.001*
	Conventional ridge split	46.14 ± 2.97	40-50			

Table 2 presents a comparison of the ISQ between tooth regions (36/46 and 37/47) within each technique: osseodensification and conventional ridge splitting. For osseodensification, the mean ISQ for teeth 36/46 was 70.57 ± 6.40, and for teeth 37/47, it was 69.43 ± 6.37, with a mean difference of 1.14 (p = 0.744), indicating no statistically significant difference between the tooth regions. In the conventional ridge split group, the mean ISQ was identical for both 36/46 and 37/47 at 46.14 ± 2.79 and 46.14 ± 2.97, respectively, with a mean difference of 0 (t = 0, p = 1), confirming no significant difference. These results indicated that both osseodensification and conventional ridge split techniques yielded consistent ISQ values across the mandibular posterior tooth regions (36/46 and 37/47). The lack of variability suggested that tooth position did not significantly influence implant stability within each technique, supporting its uniform application in these regions.

**Table 2:** Comparison of primary implant stability (implant stability quotient as ISQ) between tooth regions within each technique by independent t test. p > 0.05: non-significant, 36/46: mandibular right and left first molar teeth, 37/47: mandibular right and left second molar teeth. Data is presented in the form of mean ± standard deviation (SD).

Technique	Tooth region	Mean ± SD	Range	Mean difference	t value	p-value
Osseodensification	36/46	70.57 ± 6.40	61-82	1.14	0.335	0.744
	37/47	69.43 ± 6.37	61-82			
Conventional ridge split	36/46	46.14 ± 2.79	41-50	0	0	1
	37/47	46.14 ± 2.97	41-50			

## Discussion

Primary implant stability serves as a fundamental prerequisite for successful osseointegration, with its achievement being contingent upon multiple factors, including surgical methodology, bone density characteristics, and implant design [7]. The present investigation conducted a comparative analysis between ridge expansion via conventional ridge split and osseodensification approaches in thin posterior mandibular ridges, with quantitative assessment revealing statistically significant ISQ measurements associated with the osseodensification protocol. These empirical observations corroborate the findings of Elgrany et al. [3], whose research demonstrated that the osseodensification technique promotes increased bone mineral density adjacent to prepared osteotomy sites through controlled compaction rather than excision of osseous tissue. The mechanical superiority of osseodensification derives from its utilization of specially designed rotary instruments that employ a non-excisional, counterclockwise rotational movement pattern to achieve bone condensation [8,13]. This autologous bone compaction phenomenon results in enhanced bone-to-implant surface integration, thereby optimizing primary mechanical retention [14]. Conversely, the conventional ridge split technique necessitates complete osteotomy and subsequent lateral displacement of both buccal and lingual cortical plates, a process that may induce microfractures within the trabecular architecture and consequently compromise initial implant stabilization, particularly in regions exhibiting diminished bone density [5,6].

The osseodensification protocol offers distinct clinical advantages through its tissue-conservative nature, which maintains osseous vascular integrity while minimizing iatrogenic trauma relative to conventional ridge split procedures [15]. Although conventional ridge split remains an effective modality for achieving ridge width augmentation, the technique carries inherent procedural risks, including cortical plate fracture and impaired healing secondary to periosteal vascular compromise [16]. The biomechanical alterations induced by osseodensification-mediated bone densification may theoretically permit earlier prosthetic loading sequences, though this hypothesis requires validation through longitudinal clinical investigations [9]. Of particular interest, the current study's data analysis revealed no statistically significant variation in ISQ measurements relative to anatomical positioning (first versus second mandibular molar regions), suggesting that surgical technique selection exerts greater influence on primary stability than site-specific anatomical variations. This finding receives support from the work of do Vale Souza et al. [17], who documented a significant positive correlation between implant insertion torque values and resonance frequency analysis measurements, implying that the mechanical advantages conferred by osseodensification may manifest clinically through elevated insertion torque parameters.

The biological sequelae following implant placement demonstrate notable divergence between these two surgical approaches. The osseodensification technique appears to stimulate localized osteoprogenitor cell activation through controlled mechanical compression of trabecular bone, potentially expediting the establishment of secondary stability [18]. In contrast, ridge splitting procedures initiate a healing cascade dependent upon osseous remodelling following mechanical displacement, which may prolong the temporal progression toward secondary stabilization [19]. Peri-operative soft tissue management represents another critical consideration, as conventional ridge split protocols typically require extensive mucoperiosteal reflection with consequent increased post-operative morbidity, while osseodensification techniques frequently permit minimally invasive or flapless approaches that may reduce surgical complications [20]. However, the current body of literature contains limited comparative data regarding long-term peri-implant mucosal health outcomes between these treatment modalities.

While the present investigation demonstrates clear advantages of osseodensification in achieving primary mechanical stabilization, a comprehensive evaluation of longitudinal outcomes, including implant survival rates and crestal bone maintenance, remains imperative. Emerging evidence suggests that conventional ridge split procedures may ultimately achieve comparable secondary stability through progressive osseous regeneration despite initially inferior ISQ measurements [21].

Strengths

This randomized controlled trial was conducted with rigorous methodological standards, adhering to CONSORT guidelines, which enhances the reliability and reproducibility of the findings. The use of standardized surgical protocols, identical implant systems, and objective outcome measures, such as ISQ assessed via the Osstell Mentor device, minimized variability and ensured precise comparisons between the osseodensification and conventional ridge split techniques. Blinding of outcome assessors, data collectors, and statisticians further reduced bias, strengthening the validity of the results. The study's focus on the posterior mandible, a challenging anatomical region due to its narrow ridge and variable bone density, provides clinically relevant insights for implant dentistry. Additionally, the inclusion of CBCT for pre-operative assessment contributes to the study's scientific rigor.

Limitations and future recommendations

This study has certain limitations that should be acknowledged. The sample size, while sufficient for detecting significant differences in ISQ values, may not fully capture rare complications or long-term outcomes associated with either technique. The study was limited to a three-month follow-up period, which is adequate for assessing primary stability but insufficient for evaluating long-term osseointegration or implant survival rates. Additionally, the study was conducted in a single center, potentially limiting the generalizability of the findings to diverse populations or clinical settings. Future research should involve larger, multicenter trials with extended follow-up periods to assess the durability of implant stability and clinical success. Comparative studies exploring the cost-effectiveness and patient-reported outcomes, such as post-operative discomfort or satisfaction, could further elucidate the practical advantages of osseodensification versus ridge split techniques. Finally, investigations into the biomechanical effects of osseodensification in varying bone density types could refine its indications and optimize surgical protocols.

Additionally, patient-specific variables such as pre-operative bone density quantification (via Hounsfield unit analysis) and systemic health parameters (including metabolic bone disorders and endocrine pathologies) represent potentially significant confounding variables that were not systematically evaluated in this study. Future research initiatives should incorporate advanced imaging modalities and histomorphometric evaluation to elucidate the comparative osteogenic dynamics associated with these distinct surgical approaches.

## Conclusions

This randomized controlled trial demonstrated that both conventional ridge splitting and osseodensification techniques are viable approaches for implant placement in narrow posterior mandibular ridges. However, osseodensification consistently provided superior primary implant stability, as evidenced by significantly higher ISQ values than those in the conventional ridge split group. The technique's ability to preserve and compact the bone during osteotomy preparation likely contributed to the enhanced stability observed. Given these findings, osseodensification may offer a more predictable and biomechanically favorable alternative to conventional ridge augmentation procedures, particularly in anatomically compromised sites. Its minimally invasive nature, combined with the potential for early prosthetic loading, makes it a valuable option in clinical implantology. Further long-term studies are needed to assess its impact on secondary stability, bone remodelling, and overall implant success rates.
